# Detection and clinical characteristics analysis of respiratory viruses in hospitalized children with acute respiratory tract infections by a GeXP‐based multiplex‐PCR assay

**DOI:** 10.1002/jcla.23127

**Published:** 2019-11-27

**Authors:** Huanhuan Huang, Suqing Chen, Xiaoyan Zhang, Linliang Hong, Yongbin Zeng, Bin Wu

**Affiliations:** ^1^ Department of Pediatrics The First Affiliated Hospital of Fujian Medical University Fuzhou China; ^2^ Department of Laboratory Medicine The First Affiliated Hospital of Fujian Medical University Fuzhou China

**Keywords:** acute respiratory tract infection, children, clinical characteristics, multiplex PCR

## Abstract

**Background:**

The information regarding viral epidemiology and clinical characteristics in hospitalized children with acute respiratory tract infection (ARTI) in central Fujian is limited. In this study, we aimed at analyzing the viral epidemiology and clinical characteristics of ARTI in hospitalized children admitted to The First Affiliated Hospital of Fujian Medical University.

**Methods:**

Cohort of 386 hospitalized children (31 days to 15 years) diagnosed with ARTI admitted to the Department of Pediatrics from January 1, 2018, to December 31, 2018, was enrolled in this study. Nasopharyngeal swab or sputum samples on the day of hospitalization were tested for 11 viruses via a GeXP‐based multiplex‐PCR assay. The viral profiles and clinical characteristics were analyzed.

**Results:**

The overall positive rate of the samples was 43.26% (167/386). Among the 167 positive samples, 134 (80.24%, 134/167) had a single virus and 33 (19.76%, 33/167) had multiple viruses. There was a significant difference in the frequency of single vs mixed infections among positive samples (80.24% vs 19.76%; *χ* ^2^ = 122.168, *P* = .000) as well as among the total examined samples (34.72% vs 8.55%; *χ* ^2^ = 77.945, *P* = .000). Human rhinovirus was the most prevalent virus (17.36%, 67/386), followed by influenza A (5.96%, 23/386) and human adenovirus (5.70%, 22/386). There was no significant difference in the etiological distribution of viral pathogens between males and females (*χ* ^2^ = 0.480, *P* = .489). Viral infections were more likely to occur in the winter‐spring months than in the summer‐autumn months (52.51% vs 33.53%, *χ* ^2^ = 13.830, *P* = .000).

**Conclusions:**

The GeXP‐based multiplex PCR is an accurate and high‐throughput assay allows us to quickly detect multiple respiratory viruses simultaneously in pediatric patients. Our study provides information on the viral profiles and clinical characteristics in hospitalized children with ARTI, which would help better effective prevention strategies.

## 1 INTRODUCTION

Acute respiratory tract infection (ARTI) is a leading cause of hospitalization, morbidity, and mortality in children in pediatrics throughout the world.^1^, ^2^ Many pathogens including bacteria, virus, *mycoplasma*, *chlamydia,* and fungi can result in ARTI; virus (such as human respiratory syncytial virus [HRSV], human rhinovirus [HRV], influenza virus [IFV], human coronavirus [HCOV], and human adenovirus [HADV]) has been identified as a major cause in ARTI in children.^3^ Identifying the pathogens of viral infection timely is especially important for early diagnosis and clinical decision‐making for the pediatricians.

Presently, several diagnostic methods for the detection of respiratory virus, including virus culture, viral‐antigen detection, and viral‐antibody detection have been described.^4^, ^5^ Virus culture is a gold standard method; however, this method is labor‐intensive and time‐consuming, making it impractical to be used in the clinical laboratory.^5^ Antigen and antibody detection methods are easy‐to‐perform; however, they exhibit poor sensitivity and may have a false negative or positive reaction.^6^ In contrast, molecular techniques, such as polymerase chain reaction (PCR) and real‐time fluorescent PCR assays, are sensitive and specific for virus detection^7^; however, only one virus can be detected by conventional PCR at a time. It should be noted that multiplex‐PCR technology can simultaneously detect multiple pathogens at the same time, which is also easy‐to‐operate and need less workforce.^7^

It is well known that ARTI prevalence in children may vary in different geographic regions and different seasons.^4^, ^8^ However, information regarding viral ARTI in pediatric hospitalized children in Fuzhou city (central Fujian) is still limited. Thus, to better understand the information about the epidemiology of the pathogens in pediatric hospitalized patients with ARTI and provide effective prevention strategies, we aimed in this study to investigate the epidemiology of respiratory viruses via a GeXP‐based multiplex‐PCR assay in children under 15 years of age in pediatrics.

## 2 MATERIALS AND METHODS

### 2.1 Study design and study population

This study was conducted from January 1, 2018, to December 31, 2018, in The First Affiliated Hospital of Fujian Medical University. The inclusion criteria were as follows: patients under 15 years old, acute fever, and symptoms of ARTI. The definition of ARTI was according to diagnostic criteria of **“Zhu Futang Practical of Pediatrics”**.^9^ Briefly, patients with ARTI appear at least one of the following symptoms: sore throat, cough, shortness of breath, or coryza as an acute onset of symptoms within two days. The exclusion criteria for all participants were as follows: antiviral, antibiotic, or hormonal drug treatment prior to admission; and patients receiving radiotherapy, chemotherapy, or immunosuppressive therapy.

Demographic, clinical laboratory supporting information, imaging results, etc, were obtained from each enrolled patient. The study was approved by Ethics Review from Branch from Research and Clinical Technology Application, Ethics Committee of First Affiliated Hospital of Fujian Medical University, and carried out according to the 1975 Declaration of Helsinki. Informed consent was obtained from each subject before the enrollment.

### 2.2 Sample collection

Nasopharyngeal swab (NPS) or sputum samples were obtained from patients with symptoms of ARTI on the day of hospitalization.

### 2.3 Total nucleic acid extraction

Total nucleic acid was extracted using a nucleic acid extraction kit following the manufacturer's instruction (Ningbo ZD Biotechnology Co., Ltd).

### 2.4 Reverse transcription PCR

The 20‐μL PCR amplification reaction mixtures contained 14 μL of Premix, 1 μL of RT‐PCR reverse transcriptase, and 5 μL of nucleic acid. RT‐PCR conditions were as follows: 25°C for 5 minutes and then 50°C for 15 minutes; the reaction was terminated by incubation at 95°C for 2 minutes.

### 2.5 GeXP‐based multiplex‐PCR assay

Multiplex‐PCR conditions were as follows: step 1, 94°C for 30 seconds, 65 to 60°C touchdown PCR for 30 seconds, and 72°C for 60 seconds, repeated for six cycles; step 2, 94°C for 30 seconds, 60°C for 30 seconds, and 72°C for 60 seconds, repeated for 29 cycles; step 3, 72°C for 10 minutes; and step 4, 4°C.

The 10‐μL amplified products were added into the 287 μL loading buffer (SLS) and 3 μL SizeStandard‐400/Size 420, and then assessed using the GenomeLab Gene Expression Profiler Genetic Analysis System (Beckman Coulter). The kit of GeXP‐based multiplex‐PCR assay (Health Gene Technologies) targets 13 pathogens, including 11 kinds of virus (human bocavirus [BOCA], human adenovirus [HADV], human coronavirus [HCOV], human metapneumovirus [HMPV], human parainfluenza virus [HPIV], human respiratory syncytial virus [HRSV], human rhinovirus [HRV], influenza A virus H3N2 [H3N2], including influenza A [IFVA], influenza A H1N1[H1N1], and influenza B virus [IFVB]), 1 *chlamydia* (*chlamydia pneumoniae* [CP]), and 1 *mycoplasma* (*mycoplasma pneumonia* [MP]). The other two non‐viral pathogens (*chlamydia pneumoniae* and *mycoplasma pneumoniae*) were excluded for analysis in this study.

### 2.6 Statistical analysis

The statistical analysis was performed using statistical analysis software SPSS package version 23.0 for Mac OS X (SPSS Inc). Continuous variables were expressed as means ± standard deviations. Positive percentages in male and female patients and positive percentages among different age groups or different season groups were analyzed with the chi‐square test. A *P*‐value <.05 was considered to be statistically significant.

## 3 RESULTS

### 3.1 Confirmation of the GeXP‐based assay

**Figure 1:**
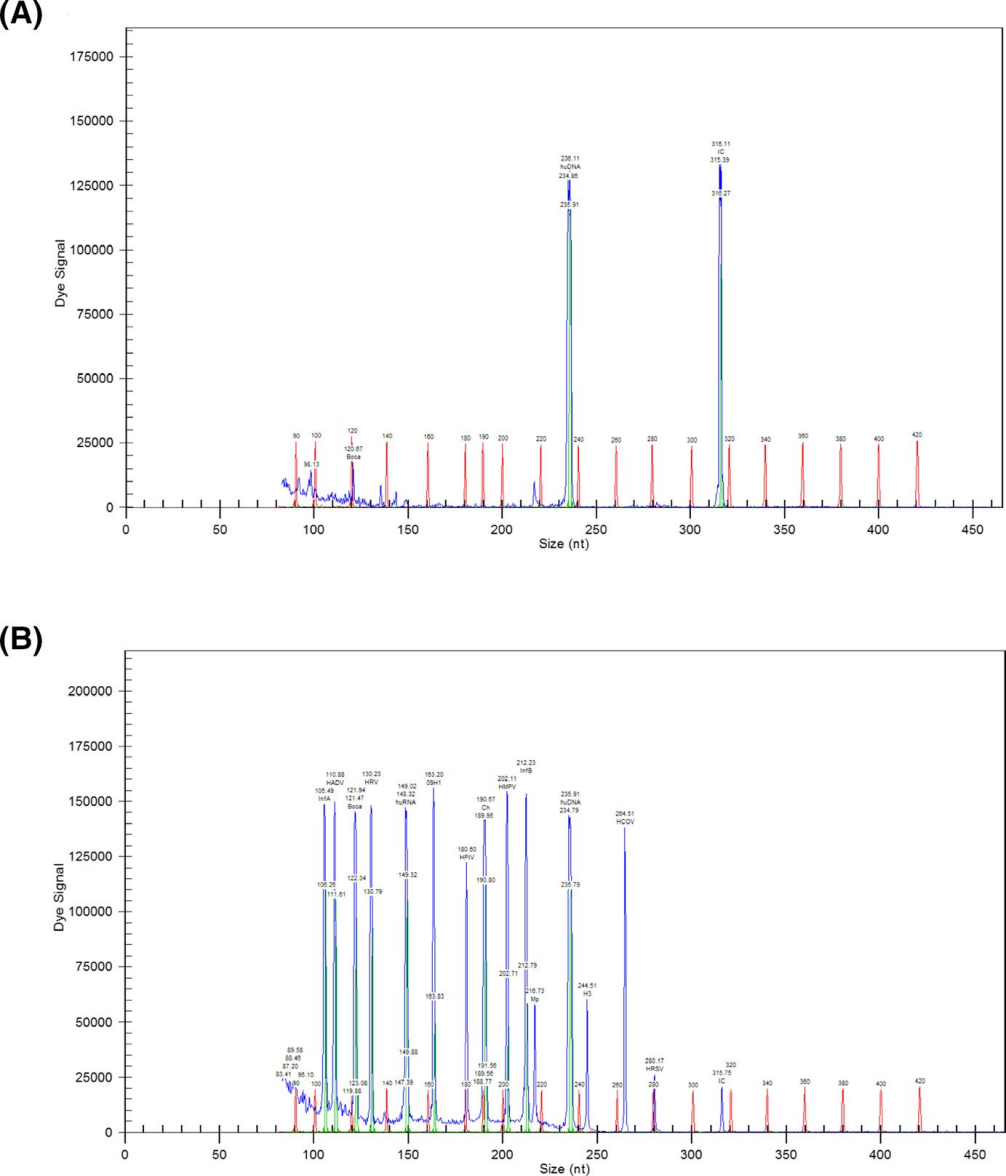
No‐template control (NTC) and positive control (PC) of the GeXP‐based assay. A, No‐template control. B, Positive control. The abscissa value indicates the size of the detected PCR product fragment, and the ordinate value represents the intensity of the fluorescent signal of the PCR product. The value of the fluorescent signal is proportional to the amount of the PCR product

RNA‐free water and the plasmid carrying the corresponding viral nucleic acid were used as a no‐template control (NTC) and positive control (PC), respectively. As indicated in Figure 1, no corresponding peak except the internal reference was observed in the NTC. Whereas 11 virus peaks appeared in the PC, and the peaks were well separated. No crossover reaction was found in PC. These indicated that the results of GeXP‐based assay were accurate and reliable.

### 3.2 Clinical characteristics of patients

A total of 386 patients diagnosed with acute respiratory tract infection, aged 31 days to 15 years, were enrolled in the present study from January 1, 2018, to December 31, 2018. Of the 386 enrolled children, 236 were boys and 150 were girls. The general characteristics of the enrolled patients were presented in Table 1.

**Table 1:** General clinical characteristics of the patients

Characteristics	Number of subjects
Total	386
Gender, No. (%)	
Male	236 (61.14)
Female	150 (38.86)
Age, No. (%)	
<1 y	96 (24.87)
1‐3 y	98 (25.39)
3‐5 y	95 (24.61)
5‐15 y	97 (25.13)
Clinical diagnosis, No. (%)	
Upper respiratory tract infection	28 (7.25)
Lower respiratory tract infection	358 (92.75)

### 3.3 Results of respiratory viruses detected overview

The overall positive rate of the samples was 43.26% (167/386). Among the 167 positive samples, 134 (80.24%, 134/167) had a single virus and 33 (19.76%, 33/167) had multiple viruses (co‐infection with two or more viruses). The detection rate of single infection and multiple infections of total analytical samples was 34.72% (134/386) and 8.55% (33/386), respectively. There was a significant difference in the frequency of single vs mixed infections among the positive samples (80.24% vs 19.76%; *χ* ^2^ = 122.168, *P* = .000) as well as among the total analytical samples (34.72% vs 8.55%; *χ* ^2^ = 77.945, *P* = .000). Human rhinovirus was the most prevalent virus (17.36%, 67/386), followed by influenza A (5.96%, 23/386) and human adenovirus (5.70%, 22/386). The positivity rates of other viruses were as follows: IFVA‐H1N1, 4.40% (17/386); HRSV, 2.85% (11/386); IFVB, 2.85% (11/386); HMPV, 1.81% (7/386); HPIV, 1.55% (6/386); and BOCA, 0.78% (3/386).

### 3.4 Age and gender distributions of the respiratory viruses

The positive rates of viral infection in male and female patients were 44.49% (105/236) and 40.67% (61/150), respectively. There was no significant difference in the etiological distribution of viral pathogens between males and females (*χ* ^2^ = 0.480, *P* = .489). In this study, all the patients were grouped by age as follows: infants (age: <1 year), toddlers (age: 1‐3 years), preschoolers (age: 3‐5 years), and school‐aged children (age: 5‐15 years). The distributions of the viral etiologies of the four age groups were shown in Table 2; however, no significant difference among the different age groups was observed.

**Table 2:** The profiles and distributions of respiratory viruses among different age groups

Characteristics	Infants (<1 y), n = 96		Toddlers (1‐3 y), n = 98		Preschoolers (3‐5 y), n = 95		School‐aged children (5‐15 y), n = 97		All ages, n = 386		*χ* ^2^	*P*
	No. Pos	Prevalence (%)	No. Pos	Prevalence (%)	No. Pos	Prevalence	No. Pos	Prevalence (%)	No. Pos	Prevalence (%)		
Positive	38	39.58	47	47.96	43	45.26	39	40.20	167	43.26	1.934	.586
Single	33	34.37	39	39.80	35	36.84	30	30.92	137	35.49	1.803	.614
Mixed	5	5.21	8	8.16	8	8.42	9	9.28	30	7.77	1.264	.738
BOCA	0	0	3	3.06	0	0	0	0	3	0.78	5.031F	.061
HADV	3	3.13	6	6.12	5	5.26	10	10.31	24	6.22	4.510	.211
HCOV	0	0	0	0	0	0	0	0	0	0	NA	NA
HMPV	2	2.08	2	2.04	3	3.16	0	0	7	1.81	3.012F	.388
HPIV	3	3.13	2	2.04	0	0	1	1.03	6	1.55	3.115F	.419
HRSV	6	6.25	3	3.061	2	2.11	0	0	11	2.85	6.93F	.059
HRV	13	13.54	17	17.35	23	24.21	15	15.46	68	17.62	4.259	.239
IH3N2	0	0	0	0	0	0	0	0	0	0	NA	NA
IFVA	4	4.17	8	8.16	5	5.26	6	6.19	23	5.96	1.491	.684
H1N1	2	2.08	6	6.12	2	2.11	7	7.22	17	4.40	4.681	.183
IFVB	4	4.17	3	3.06	3	3.16	1	1.03	11	2.85	1.939	.624

Abbreviations: BOCA, human bocavirus; H1N1, influenza A H1N1; H3N2, influenza A virus H3N2; HADV, human adenovirus; HCOV, human coronavirus; HMPV, human metapneumovirus; HPIV, human parainfluenza virus; HRSV, human respiratory syncytial virus; HRV, human rhinovirus; IFVA, Influenza A; IFVB, influenza B virus; NA, not applicable.

### 3.5 Seasonal distribution of the respiratory viruses

The hospitalized children were divided into four groups according to the seasons: (a) spring group (March, April, and May), 149 cases; (b) summer group (June, July, and August), 108 cases; (c) autumn group (September, October, and November), 59 cases; and (d) winter group (December, January, and February), 70 cases. The virus detection rates for different seasons were shown in Table 3. Overall, the positive detection rate was 50.33%, 36.11%, 28.81%, and 57.14% in spring, summer, autumn, and winter, respectively. Viral infections were more likely to occur in winter‐spring months than in the summer‐autumn months (52.51% vs 33.53%, *χ* ^2^ = 13.830, *P* = .000). It should be noted that HADV occurred more frequently in summer. HRSV was detected almost throughout the year. Other viruses occurred almost sporadically throughout the year without obvious seasonal trends.

**Table 3:** Seasonal distribution of respiratory viruses detected by GeXP‐based multiplex‐PCR assay

Pathogen	Spring (149)		Summer (108)		Autumn (59)		Winter (70)		*χ* ^2^	*P*
	No. Pos (n)	Prevalence (%)	No. Pos (n)	Prevalence (%)	No. Pos	Prevalence (%)	No. Pos	Prevalence (%)		
BOCA	1	0.67	1	0.93	1	1.695	1	1.405	1.386F	.831
HADV	5	3.36	12	11.11	3	5.08	4	5.71	6.113F	.098
HCOV	0	0	0	0	0	0	0	0	NA	NA
HMPV	4	2.68	1	0.93	1	1.695	1	1.43	1.126F	.87
HPIV	3	2.01	1	0.93	1	1.695	1	1.43	0.787F	.94
HRSV	5	3.36	2	1.85	1	1.695	3	4.29	1.299F	.758
HRV	27	18.12	22	20.37	9	15.25	10	14.29	1.352	.724
H3N2	0	0	0	0	0	0	0	0	NA	NA
IFVA	14	9.40	0	0	0	0	9	12.86	22.229F	**.000**
H1N1	11	7.38	0	0	1	1.695	5	7.14	11.608F	**.005**
IFVB	5	3.36	0	0	0	0	6	8.57	11.215F	**.004**
Total	75	50.33	39	36.11	17	28.81	40	57.14	15.548	**.001**

Note

The value in bold indicated a significant difference (*P* < .05) performed by multiple comparison.

Abbreviations: BOCA, human bocavirus; F, Fisher's exact test; H1N1, influenza A H1N1; H3N2, influenza A virus H3N2; HADV, human adenovirus; HCOV, human coronavirus; HMPV, human metapneumovirus; HPIV, human parainfluenza virus; HRSV, human respiratory syncytial virus; HRV, human rhinovirus; IFVA, Influenza A; IFVB, influenza B virus; NA, not applicable.

## 4 DISCUSSION

The respiratory virus is an important cause of acute respiratory infection in children. After an acute respiratory infection occurs in children, the specific pathogens are often unclear and can be easily misdiagnosed or ignored by clinicians, resulting in the abuse of antibiotics and delayed treatment. Consequently, it is necessary to early detect the specific pathogen, as well as understand the pathogenic spectrum and epidemic features of acute respiratory infection in children in particular regions.

Currently, nucleic acid amplification techniques (NAATs) (such as PCR and multiplex PCR) have been increasingly used for identification of pathogens in infectious respiratory diseases^7^; and one of the multiplex‐PCR technology, GenomeLab gene expression profiler genetic analysis system (GeXP)‐based assay (developed by Beckman Coulter), has been adopted in the infectious pathogens diagnostics^10^, ^11^, ^12^; this detection technique has been applied in clinical for over 5 years; however, more clinical application studies are still necessary, especially in southeast China. GenomeLab gene expression profiler genetic analysis system is a high‐sensitive and specific analytical platform for high‐throughput nucleic acid detection based on capillary electrophoresis separation technology, multiple gene analysis technology, and high‐sensitivity laser‐induced fluorescence technology.^10^ The GeXP‐based multiplex‐PCR assay can easily detect and analyze up to 30 genes simultaneously in a single analysis by converting amplification with multiple primers to amplification with a pair of universal primers.^10^

In this study, we used GeXP‐based multiplex‐PCR assay to detect 11 kinds of virus, as well as *mycoplasma pneumonia* and *chlamydia pneumoniae* in 386 samples from hospitalized children with acute respiratory tract infection over a period of 1 year. It was a rapid, accurate, and high‐throughput analytical method which can finish the detection process within 4 hours and may aid the pediatricians for early medical decision‐making.

Overall, the positive rate for the detection of viruses in the present study was 43.26%; HRV, IFVA, HADV, and H1N1 ranked top four in all the infected viruses. With the application of this multiplex‐PCR assay, the detection of multiple co‐infecting viruses had become common which reflected the high sensitivity of this multiplex‐PCR assay. Kouni S described that the prevalence of viral co‐infections ranged between 15% and 44% in children seen in the hospital or emergency department.^13^ In the present study, the detection rate of multiple viral infections among children with positive samples was 19.76%, the largest proportion of which included IFVA/IFVA‐H1N1. However, whether multiple viral infections are associated with a more severe clinical presentation compared with a single infection remains controversial and still need further study.^13^

In our study, gender had no influence on the detection rate of respiratory viruses, which was consistent with other reports.^8^, ^14^, ^15^ Many studies documented that respiratory viral infection was characterized by seasonal distributions, with their peaks lasting from winter to early spring.^15^, ^16^ In the present study, the detection rate of respiratory viruses was also higher in winter and spring. One possible reason was that climate change may be an important factor contributing to the variability in seasonal trends and viral etiologies of ARTI; the cold weather during this period may increase the risk of respiratory infections. When the analysis was carried out according to the ages, we found that the total virus detection rate and virus prevalence of different age groups were not significant different. However, in the study of Li et al, though no significant differences in the positivity rate of the different age groups were observed, the prevalence of viruses was different between different age groups. This differences in the prevalence of viruses in different age groups observed between our and Li's study may be due to the following:first, the relatively small sample sizes of our study; and second, the differences in the sensitivity of these two analytical assays (GeXP and FilmArray) used in the studies.

Influenza occurs all over the world, with an annual global attack rate estimated at 5%‐10% in adults and 20%‐30% in children.^17^ Influenza virus infection (IFVI) can cause primary viral pneumonia, which may progress to a potentially fatal outcome. It is documented that influenza causes significant morbidity in young children and, each year, leads to hospitalization of approximately 870 000 children under 5 years of age worldwide.^18^, ^19^ Influenza outbreaks usually occur during the winter and spring seasons, and in our study, influenza viruses (IFVA + IFVB+IFVA‐H1N1 + IFVA‐H3N2) (51/386, 13.21%) were the second most frequently detected viruses with remarkable seasonal distributions in winter and spring months.

However, there are several limitations to our study. First, all data for this study were obtained from a single center and may not be representative of the entire pediatric population. Second, the sample size was relatively small, and more samples are needed to confirm these observations in future.

In summary, the GeXP‐based multiplex‐PCR assay allows us to quickly detect multiple respiratory infections caused by viruses. The array can detect 11 respiratory viruses simultaneously in only 4 hours. Our study provides information on the viral profiles and clinical characteristics in hospitalized children with ARTI, which would help better effective prevention strategies.
